# Information flow and protein dynamics: the interplay between nuclear magnetic resonance spectroscopy and molecular dynamics simulations

**DOI:** 10.3389/fpls.2015.00306

**Published:** 2015-05-05

**Authors:** Nina Pastor, Carlos Amero

**Affiliations:** ^1^Laboratorio de Dinámica de Proteínas y Ácidos Nucleicos, Centro de Investigación en Dinámica Celular, Universidad Autónoma del Estado de Morelos, Cuernavaca, Mexico; ^2^Laboratorio de Bioquímica y Resonancia Magnética Nuclear, Centro de Investigaciones Químicas, Universidad Autónoma del Estado de Morelos, Cuernavaca, Mexico

**Keywords:** allosterism, folding intermediates, dynamics, binding, aggregation

## Abstract

Proteins participate in information pathways in cells, both as links in the chain of signals, and as the ultimate effectors. Upon ligand binding, proteins undergo conformation and motion changes, which can be sensed by the following link in the chain of information. Nuclear magnetic resonance (NMR) spectroscopy and molecular dynamics (MD) simulations represent powerful tools for examining the time-dependent function of biological molecules. The recent advances in NMR and the availability of faster computers have opened the door to more detailed analyses of structure, dynamics, and interactions. Here we briefly describe the recent applications that allow NMR spectroscopy and MD simulations to offer unique insight into the basic motions that underlie information transfer within and between cells.

## The Importance of Motion for Information Flow

Signal transduction is firmly rooted in the interactions of proteins with diverse ligands, and in the molecular consequences of these interactions. In the past few years we have accrued a large amount of structural information on how proteins look before and after they bind select ligands, as can be seen in the protein data bank (PDB) Databases (Bernstein et al., 1977). As noted by many, this is only part of the story, though. The situation is akin to attempting to reconstruct a full ballet performance from a few still photographs of a dancer. It becomes clear that in order to understand and appreciate the choreography of a living cell and its interactions with its surroundings, dynamic information at the molecular and atomic level is essential. This is the territory of nuclear magnetic resonance (NMR) and of simulations of molecular structures, in particular molecular dynamics (MD), because of their ability to describe both structure and dynamics with atomic resolution in timescales ranging from ns to ms and longer. This review is focused on recent examples of successful combinations of NMR and MD to study proteins, as they perform their dances alone and with their ligands.

**FIGURE 1:**
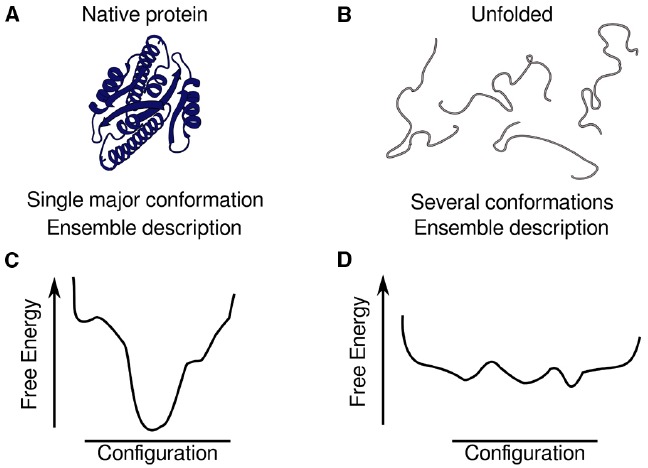
**Depiction of the ensembles of structures by conformational landscapes, with the location of minima, their depths and the heights of the barriers.** Schematic representation of ensembles of structures for a globular, natively folded protein **(A)** and an intrinsically disordered protein **(B)**. The description of a globular protein is based on an ensemble of structures clustered around a deep global minimum that can have substates in it **(C)** but represent basically the same topology, whereas the description of an IDP is based on a dynamic ensemble of different conformations represented by a collection of minima with similar depths **(D)**.

The description of the native state has shifted from single structures to that of ensembles of structures. It is therefore of interest to describe the conformational landscape of proteins, with the location of minima, their depths, and the heights of the barriers separating these minima (Figure 1). The relative depths relate to the populations of the structures corresponding to the minima, and the barriers speak of the interconversion timescale. It has been suggested that the whole conformational landscape is subject to evolution, as the relative populations and the exchange rates among possible states are all relevant for function (Teilum et al., 2011; Nussinov and Tsai, 2013) and for the proper folding *in vivo* in a biologically relevant time (Braselmann et al., 2013). Regarding protein folding, the goal is to describe the structures along the folding landscape, in order to understand the folding process and its associated diseases. These diseases result from loss of function because the protein never reaches the functional state, or because of toxic gain-of-function structures (such as amyloid fibers and oligomers) accessible from accumulated intermediates or unfolded structures (Bemporad and Chiti, 2012; Neira, 2013; Vendruscolo and Dobson, 2013; Knowles et al., 2014). Folding and aggregation intermediates are high-energy species, with low populations and fleeting lifetimes, and therefore requiring ingenious experiments and simulations to be characterized (Lee and Goto, 2012; Lane et al., 2013; Neira, 2013; Rennella and Brutscher, 2013). The correct description of the denatured state ensemble (DSE) and its residual structure is also an active subject of inquiry, as this ensemble holds clues to how folding is initiated (Bowler, 2012), probably being biased toward the topology of the native state.

Conformational landscapes are a useful tool to frame mechanistic questions regarding protein response to ligands and post-translational modifications (PTMs), going from the lock-and-key model to induced fit, conformational selection, and population shift (Changeux, 2013). An important issue in these models is whether all relevant minima pre-exist in the absence of ligand or PTM, and hence these modifications and ligands select conformations, or whether the ligand deforms the conformational landscape creating new minima as in induced-fit. The discussion in terms of a collection of states implies that altering the population of one minimum affects all others, through the Boltzmann distribution, leading to population shifts (Motlagh et al., 2014). It also highlights the importance of having enough of the protein correctly folded so that the population of functional states is physiologically adequate (Tsai and Nussinov, 2014). In order to figure out if conformational changes happen before binding or afterward, the rates of each process must be measured and compared to the binding rate. This comparison requires an explicit binding model, such as the formation of a transient encounter complex between particular states of the receptor and ligand, followed by a recognition complex and finally an adapted complex (Teilum et al., 2011), each of which can be probed independently to test the model. Interestingly, as a consequence of detailed balance, a process that uses induced-fit while binding will result from conformational selection in unbinding and *vice versa* (Weikl and Paul, 2014).

Native folded states are not the only players in signaling cascades in the cell, as the studies on intrinsically disordered proteins (IDPs) and intrinsically disordered regions (IDRs) in folded proteins have shown (Liu and Huang, 2014; Uversky, 2014; Wright and Dyson, 2014). These proteins are conformationally and dynamically heterogeneous, in dynamic equilibrium among multiple conformational states separated with low barriers (Figure 1). The issue in binding of IDPs/IDRs is that these chains have a lot of conformational entropy, and may lose much of it upon binding. There are various strategies to compensate for this entropy loss. One option is a favorable enthalpy and dehydration, resulting in weak but specific binding (Flock et al., 2014). Therefore, the amount of lost entropy can be used to tune function, and some complexes retain high flexibility, leading to the concept of ligand clouds (Jin et al., 2013). Residual structure can be stabilized upon binding, reducing the entropy cost, or favor the unbound state (Wright and Dyson, 2014). Another option is to use extended regions of a few amino acids as binding sites, so again the entropy cost is minimal, and also keep high flexibility in the connecting loops. Yet another strategy is to have multiple degenerate binding motifs, hence the combinations increase entropy and the cost is lower. IDRs can be pre-fixed into productive conformations by PTMs or other extrinsic factors. These extrinsic factors can be used as coincidence detectors in IDPs, giving rise to all or none responses, graded responses or molecular clocks (Wright and Dyson, 2014).

The actual structures in the local minima of these landscapes and the transformations, structural and/or dynamic, that proteins undergo upon ligand binding are the heart of catalysis (McGeagh et al., 2011), allostery and information flow (Nussinov and Tsai, 2013). Structural flexibility, reflected in the local and global motions of backbone and side chain atoms (Motlagh et al., 2014), can be either increased or decreased upon ligand binding, as the ligand opens or closes energy and information transfer pathways in the protein, affecting the packing or even the folding of the structure (Marsh et al., 2012). These changes in flexibility, together with changes in translational and rotational freedom of the ligand and the protein, are the entropic component of the free energy of binding, while the direct interactions with the ligand and the cost or gain in energy of the associated conformational change contribute to the enthalpy. It may well happen that the flexibility responsible for the stability of the ground state of the protein is orthogonal to the dynamics required for catalysis (Teilum et al., 2011). Each protein-ligand complex displays a particular balance of enthalpy and entropy, and therefore, an associated molecular mechanism. The role of entropy in complex formation is one of the reasons hindering a direct connection between the structure of a complex and its binding affinity (Kastritis and Bonvin, 2013).

**FIGURE 2:**
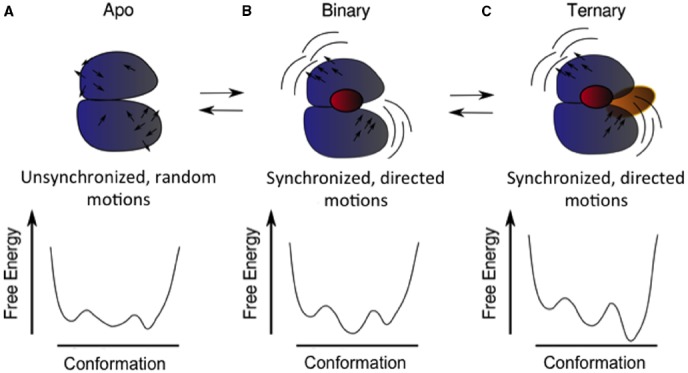
**Schematic of structural and dynamic coupling between different functional sites in a protein and how this coupling could modulate the energy landscape.** The apo or ligand-free protein **(A)** samples three conformations in its native basin and displays unsynchronized and undirected motions (black arrows). Upon binding of the first ligand **(B)**, a particular conformation is selected (the minimum in the middle of the landscape), and also the motions of the two main lobules of the protein become synchronized (notice the similar direction of the arrows in each domain). These motions favor the binding of the second ligand **(C)**, selecting another of the possible conformations.

Many eukaryotic proteins are composed of more than one domain, connected by linkers. The role of linkers in information transfer between domains goes beyond a reduction in sampling space; some authors (Ma et al., 2011) have suggested that linker sequences have propagation pathways pre-encoded in them, favoring particular relative orientations between domains, so information transfer is more efficient than what would be achieved by a random walk. The search for the information transfer pathways across and within proteins is a fertile area of research (Nussinov and Tsai, 2013, 2014b; Feher et al., 2014). These involve structural and/or dynamic coupling between functional sites (Figure 2), and may be composed of multiple routes crisscrossing the protein (Tsai and Nussinov, 2014), suggesting that most of the protein surface is a candidate as an allosteric site. The outcome of information transfer is to stabilize selectively a particular conformation of those thermally accessible to the protein.

An interesting corollary is that each conformation or dynamic state accessible through flexibility could be associated to a different function, selected by particular ligands, leading naturally to selectivity even in the presence of promiscuous interactions at a single or multiple sites: each binding event promotes particular structural and/or dynamic changes in the protein, explaining how small changes in the ligand can transform it from an agonist to an antagonist (Nussinov and Tsai, 2014b). Selectivity is important, as it allows for multiple “conversations” to take place simultaneously in the cell without scrambling the messages. An intriguing consequence of the paradigm of population shift is that all that is needed for signal transmission is to select the appropriate conformations of the receptor and the ligand; the affinity controls the lifetime of the complex, not the message that is sent (Nussinov and Ma, 2012). Proteins emerge from these analyses as integrators of signals (Nussinov and Tsai, 2014a), where their non-linear output depends on the order and location in which ligands and/or PTMs are bound or acquired, becoming logical gates as part of the complex circuitry of life (Nussinov et al., 2013). Understanding how these effects come about is important, among other things, for tailored drug design both at active sites and at allosteric sites (Nussinov and Tsai, 2014b, 2015).

All of the previous discussion hinges on being able to see both the structures and how they change in time in response to ligands or other perturbations, so the proposed mechanisms can be validated or discarded. The examples we discuss below are aimed at the atomic-level exploration of the conformational landscapes of both folded proteins and IDPs relating to catalysis, binding and signaling, allostery, folding, and aggregation.

## MD and NMR Toolkit to Study Protein Dynamics

The molecular complexity of proteins and the span in timescales of their dynamics make their study a daunting task, achievable only through the use of multiple approaches, both experimental and computational. This review is centered in the joint application of NMR and MD simulations to understand information flows in biology. Another computational tool that has been extensively used to study protein dynamics is the elastic network model (ENM), which explores fluctuations around the native structure, and has been used to explain allosteric regulation and conformational changes. We suggest a couple of recent reviews (Dehouck and Mikhailov, 2013; Bastolla, 2014) for the combination of ENM and NMR, for interested readers.

### Molecular Dynamics Simulations

Molecular dynamics simulations work as “molecular microscopes” (Dror et al., 2012), yielding physically sensible ensembles of molecular structures, linked temporally (see, for example McGeagh et al., 2011). Carrying out MD simulations has become relatively straightforward, with tutorials and web servers that help both experts and newcomers to set up and run the simulations (Cui and Nussinov, 2014). The net result of an MD simulation is a large collection of structures of the protein of interest, at ps or sub-ps resolution, from which any observable property that can be cast in terms of atomic coordinates can be calculated and compared to experimental data. This translates to following the sequence of events that leads to function at atomic resolution under ideal conditions: an accurate, experimentally validated force field and sufficient sampling (Elber, 2011).

**FIGURE 3:**
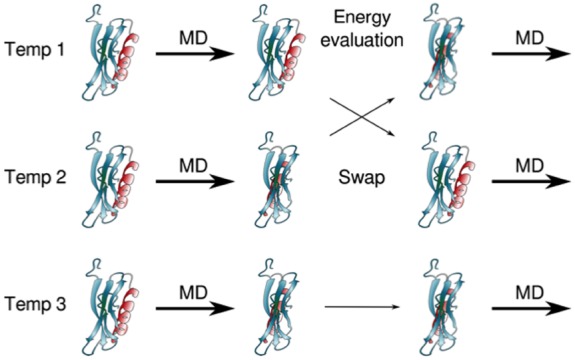
**Example of an enhanced sampling method consisting on multiple MD simulation replicas at different temperatures (each row).** At defined time intervals, which can vary typically from a few to hundreds of ps, the energy of the structures of each replica are evaluated. If the energy of run 1 is lower than that of run 2, or if it is higher but allowed by the Boltzmann factor, the replicas are exchanged, so now the structure from run 1 is simulated at temperature 2, and the structure from run 2 is simulated at temperature 1 (exchange between the top and middle rows). Otherwise, as shown for temperature 3 (lower row), there is no exchange and run 3 continues to be simulated at temperature 3. Exchanges are attempted until all replicas have been run at all temperatures. Structures are then collected as a function of temperature, and data can be analyzed for all temperatures, or only for a particular temperature, of functional or physiological relevance.

The sampling issue has made it complicated to escape the anecdotal description of folding and binding processes. A couple of strategies to improve sampling in unbiased full atomic detail simulations are distributed computing using maximum parallelization in the generation of tens or hundreds of thousands of relatively short runs (reaching tens of ms of cumulative sampling for a collection of proteins) followed by the construction of Markov state models (MSMs) to represent the network of protein conformations (reviewed in Chodera and Pande, 2011; Lane et al., 2013; Chodera and Noé, 2014), and the use of special purpose machines such as ANTON (Shaw et al., 2009) for the generation of single very long runs [up to 1 ms for bovine pancreatic trypsin inhibitor (BPTI), for example]. An alternative to studying protein folding is to follow unfolding by high temperature, assuming microscopic reversibility. These studies aim to identify the transition state ensembles (TSE) and intermediates, and to describe the DSE with its residual structure (van der Kamp et al., 2010; Toofanny and Daggett, 2012). Enhanced sampling methods are also available, such as replica exchange MD (REMD; Figure 3) using temperature or other biases as in metadynamics (Granata et al., 2013), and accelerated MD (AMD), where an energy boost is added to make minima shallower and barriers easier to jump. The simulation of IDPs/IDRs is particularly problematic (Esteban-Martin et al., 2012), because recognition and binding of these proteins happen in the μs to ms timescale, making explicit-solvent all-atom unrestricted MD a poor tool, so a collection of alternatives is presented, from coarse-grained and multi-scale models (reviewed in McGeagh et al., 2011; Nussinov, 2014; Zhou, 2014), Go models, implicit solvent simulations (Kleinjung and Fraternali, 2014) or targeted MD (Baker and Best, 2014).

Very long simulations have also been used to validate and refine the underlying physical models of the force fields used in the simulations (Esteban-Martin et al., 2012; Piana et al., 2014). Most force fields currently used are capable of describing protein folding, including folding times and stability, and also of describing structure and dynamics of native state ensembles, with errors commensurable with experimental uncertainty (Fisette et al., 2012). The description of the DSE remains problematic, as simulated ensembles are more collapsed than what experiments suggest. One potential solution to both the inaccuracy of force fields and the limitations in sampling is to add experimental restraints to the simulations, as extra terms to the force field, in a protein-specific fashion. This approach serves to define the minimum structural heterogeneity that is needed to reproduce the experimental data, both for folded and unfolded proteins. The NMR data used for this are residual dipolar coupling (RDC), chemical shift anisotropy, and distance restraints from nuclear overhauser effect (NOE) or paramagnetic relaxation enhancement (PRE) experiments. The main issues are how to apply the restraints (over single structures or over the ensemble, over primary data or secondary data, at all times or averaging after certain times; Allison, 2012; Allison et al., 2012; Esteban-Martin et al., 2012; Romo and Grossfield, 2014).

### Nuclear Magnetic Resonance Spectroscopy

Nuclear magnetic resonance provides experimental information on structure and dynamics (amplitudes and rates) of macromolecules [up to 100 kDa for structure and 1 MDa for other studies (Foster et al., 2007)]. The resonance frequency of nuclei is sensitive to their electronic environment and to their orientation with respect to an external magnetic field, and therefore to changes in conformation happening over many timescales (Kleckner and Foster, 2011; Case, 2013). These chemical shifts effects can be calculated from the nuclear coordinates, and *vice versa*, where the proper calculation should be carried out with quantum mechanics; nevertheless good empirical approximations are available in programs such as SHIFTX+, SPARTA, and camshift (reviewed in Case, 2013). The possibility to calculate chemical shifts from coordinates allows for the use of these data to constrain MD simulations.

Protein dynamics is studied with NMR by measuring relaxation rates (reviewed in Bieri et al., 2011; Kleckner and Foster, 2011; Osawa et al., 2012), spanning timescales from ps to hours or more, covering the range of motions important for information flow. The shorter the timescale, the smaller the group of atoms involved in the motion, in general. Faster motions correspond to wiggling within a substate, while slower motions involve jumping between substates. Two types of flexibility can be defined (Kleckner and Foster, 2011), static flexibility, which refers to the population of each substate in the conformational landscape, and dynamic flexibility, which refers to the rates and pathways connecting these substates. The internal dynamics of the protein causes fluctuating magnetic fields, and this alters the relaxation rates of the nuclei. These fields are the Fourier transform of time-correlation functions of distances and angles that can be calculated from MD simulations that have reached equilibrium. For the analysis, the rotation of the protein as a whole and the internal dynamics of the nuclei of the protein can be separated if they occur in different timescales. This separation is actually tricky, and is one acknowledged source of discrepancy between experimental and calculated data (the different approaches to obtain S^2^ are reviewed in Fisette et al., 2012; Allnér et al., 2015). Two-state models are the simplest used to interpret chemical exchange, where a nucleus is exposed to two different chemical environments. The intensity of the peaks reports on the population of the states generating those signals, and the width of the peaks (linewidth) reports on the relaxation rates. Relaxation dispersion experiments can yield structural, dynamic, and thermodynamic data on “invisible” states, which are populated up to a few percent and cannot be directly detected (Kleckner and Foster, 2011). These states embody the conformational selection and population shift paradigm.

Orientational information can be obtained from RDCs in weakly oriented samples. RDCs depend on the shape of the molecule, therefore as long as changes in shape are minimal, MD simulations can be used successfully to back-calculate them. Problematic areas are turns, loops, side chains, and interdomain motions. Meanwhile, paramagnetic NMR spectroscopy (Hass and Ubbink, 2014) can be useful to obtain long-range restraints for positioning and orienting molecules in a complex, or to probe residual structure by using PRE effects.

Nuclear magnetic resonance spectroscopy is also an excellent tool to study binding interactions over a wide range of affinities (from mM to high affinity), and to follow protein folding as it happens, either by fast data acquisition of heteronuclear single quantum coherence or correlation (HSQC) experiments or by H/D exchange (Bieri et al., 2011). Complex formation is followed by changes in the intensity and chemical shift of the resonances of each residue, giving not only information on the existence of the complex, but also on the details of the interface and the perturbation on the structure, as chemical shift changes can be seen not only at the interface, but also at residues located far from the binding site. NMR allows for the characterization of encounter complexes, something that cannot be done with other spectroscopic or crystallographic techniques. Also NMR relaxation data can be used to study the entropy changes that take place upon binding and recognition (Wand, 2013; Allnér et al., 2015). Last but not least, solid state NMR (ssNMR) has become a useful tool to study both structure and dynamics of membrane proteins and large aggregates, such as amyloid fibers.

### NMR and MD

**FIGURE 4:**
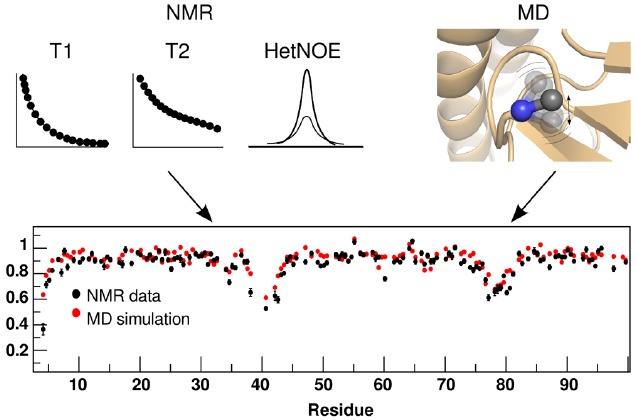
**Correspondence of dynamic parameters measured by NMR and back-calculated from an MD trajectory.** The square of the backbone order parameter (S^2^ shown in the bottom panel), which provides information about the amplitude of the motion of NH bonds or CH bonds, can be calculated from measured experimental parameters T1, T2, and HetNOE (black dots in the bottom panel), and from the MD trajectories (red dots in the bottom panel). The resulting values, for both approaches, can be compared directly.

Given the low sensitivity of NMR, there is extensive time and ensemble averaging in the data, a feature to be contrasted with the single molecule character of most MD simulations. The main idea of a combined study is to obtain from the MD simulations the underlying distribution that gives the average value obtained in the NMR experiment (Figure 4). This is a non-trivial matter due to the non-linear and multiple-valued relationships between NMR observables and protein structure. Also, it is conceivable that many different distributions can result in the same average value, so care must be taken to look at a large collection of properties, preferably with different types of averaging.

Also, the sampling limitations of the MD runs must be taken into account, as all the substates and their relative populations should be well described. Proper accounting of the relevant timescales is essential when validating and/or restraining MD simulations with NMR data (Allison, 2012; Allison et al., 2012). Short simulations (sub-μs) are adequate to reproduce S^2^ order parameters, which relate to ps–ns motions (Figure 4), but not for slower relaxation measures. Apparently, single runs of at least 10 μs are needed to achieve good agreement with RDCs and J-couplings (Allison, 2012; Esteban-Martin et al., 2012).

On the one hand we have MD simulations providing atomic detailed explanations of protein motion, for limited timescales, and on the other hand there is NMR spectroscopy, yielding information on changes in the environment of key nuclei in the proteins, related to the same motions. MD simulations can help in the interpretation of NMR data, through the generation of physically plausible conformational ensembles and the molecular mechanisms connecting the substates in these ensembles. The simulations can also supplement structural information, and point to inconsistencies in the data, as all NMR observables can be written in terms of nuclear coordinates (with care, as described above, because of the approximations involved in the models and equations that are used, and the limitations of sampling in the simulations). The collaboration of these two approaches brings simulations into the real world, and provides clear and testable molecular mechanisms for the spectral features measured by NMR, getting us closer to understanding the molecular basis for life processes.

We describe the result of the improvement on both the NMR and MD fronts over the past three years, as it pertains to information flow in biological systems involving proteins in particular. This is by no means an exhaustive account on all published work on MD or NMR, and builds on decade-long efforts by many groups. We apologize to the colleagues whose work we could not include due to space or scope limitations.

## Unrestrained MD Simulations Exploring the Conformational Landscape

These simulations are the easiest to set up and carry out, and assume that the force fields are sufficiently accurate to describe both protein structure and dynamics; ingenuity is manifested in careful data analysis, and in the biological insights that can be obtained from these runs. Most of the stories deal with the dynamics of folded proteins, alone or in complexes, as these relate to catalysis or ligand binding, and are compared to amide and methyl order parameters derived from relaxation data, dispersion experiments, and H/D exchange. The biological problems that are explored by these works range from residual structure in the DSE of an acid-unfolded protein, the effect of crowders on the native structure, identification of allosteric pathways, excited states and aqueducts, characterization of loop motions important for complex formation or catalysis, selectivity due to dynamics, and fibril stability.

Simulations of free proteins look at the intrinsic structural plasticity and at the timescales of the motions, to relate them to function. Two papers study the dynamics of BPTI (Xue et al., 2012; Persson and Halle, 2013), by analyzing the 1 ms run carried out in ANTON (Shaw et al., 2010). Because one must typically run 10 times as long as the longest lived process one wishes to study, this landmark simulation allows access up to the 100 μs range. In the first paper, Xue et al. (2012) study the native basin of BPTI, comparing the experimental relaxation dispersion data to those derived from the autocorrelation functions for individual spin states in the simulation. They identify nine states and their exchange network, linking the observed exchange rate to the 10–100 μs isomerization of one of the disulfide bonds, which results in the motion of two proximal loops involved in the inhibition of trypsin. Neither the flipping of an aromatic ring nor the slow exchange of bound water could be gleaned from the crystal structure, highlighting the relevance of dynamic studies. Despite this success, the authors acknowledge that the populations are overestimated, and the correlation times are underestimated. This is ascribed to limitations in the force field and in sampling. In the second paper, Persson and Halle (2013) followed four cavities of BPTI and the water molecules in them by deuterium and ^17^O magnetic relaxation dispersion experiments. The simulation reproduces the experimental mean survival times at the four cavities, in the range of 2–5 μs. They find single-file water chains that form transient aqueducts through tunnels or pores with lifetimes under 5 ns. These tunnels are mechanically linked to the conformation of the same disulfide bond described by Xue et al. (2012; C14–C38). Another long simulation carried out in ANTON is the 71 μs run for the active receiver domain of NtrC (Villali et al., 2014), a single chain allosteric model activated by phosphorylation. This is a case of guilt by association, because crystal structures of active and inactive states of other members of the same family had suggested that activation depended on the rotameric state of an aromatic residue (Y101) and the position of a nearby T or S (T82), and that these depend on the phosphorylation of the nearby D that is the substrate of a kinase. The main point of this study was to measure the timescale of this structural change and compare it to the response time of the whole domain, both in simulations and with NMR dispersion experiments. They find that rotamer changes are much faster than activation/inactivation, so they are not correlated to the activation process nor are they rate limiting.

Continuing with receptors and ligands, 16 ephrin receptors and their nine ligands are an intriguing example of how the same scaffold can allow for specificity (Huan et al., 2013), the binding surface is always the same, but the difference lies in the conformation of loops that line this surface. They study the EphA5 ligand-binding domain (LBD), that in the absence of ligand looks like other LBDs already bound to ephrins, just as the conformational selection model would suggest. It has an open pocket characterized by H/D exchange NMR, the absence of relaxation dispersion, and backbone relaxation indicating that these loops are very flexible in the ps–ns timescale, ideal for MD studies. This pocket is avid for a specific peptide that all other LBDs ignore, as shown by NMR titrations. Three independent 30 ns MD simulations coincide with the structural and dynamic signatures, and offer an explanation for the peculiar behavior of EphA5 compared to EphA4, a sibling receptor with the opposite dynamic behavior (motion in the μs–ms regime, closed unbound state), the electrostatic repulsion at the tips of the binding-site loops in EphA5, both of which have negative charges; in EphA4, one of the loops is neutral, allowing for stable favorable interaction between these loops. Another scenario is exemplified by the Rho-GTPase binding domain (RGB) of plexin-B1, where one small domain uses partially overlapping surfaces to interact with different partners along a signaling cascade. Zerbetto et al. (2013) analyze ^15^N relaxation data with two different approaches, and also carry out four MD simulations of 55 ns each for the wild type inactive dimer, a mutant monomer, and an active complex with Rac1. Three loops are crucial for allostery, and two possible allosteric pathways emerge from their analysis, one for dimerization and the other for GTPase binding, sharing a beta strand at their intersection. Analyzing the changes in dynamics they show that binding at loop three makes it more rigid, but this loss of entropy is gained elsewhere, at the termini of adjacent strands. The nature of protein motions and their response to ligands is an issue of practical importance for the design of inhibitors. Meli et al. (2014) analyze the interaction of a small inhibitor called sm27 with the fibroblast growth factor FGF2 at a flat interface. The inhibitor impairs binding to the FGF receptor FGFR1 both sterically and allosterically. They carried out MD simulations of 1 μs for the apo and the holo structures, as well as the NMR characterization in terms of structure, dynamics, and hydration. In order to contend with the limited sampling, they parse the μs-long MD runs into 100 ns blocks, and keep those that yield observables closer to the experimental data.

A particular case of protein–protein complexes is the amyloid fiber. Two examples showcasing the interplay of ssNMR and MD involve a prion (Lange et al., 2009) and a peptide derived from insulin (Matthes et al., 2014). The prion is HET-s, and the study involved amino acids 218–289. Its structure was previously known from ssNMR and was used as the starting structure for short (10 ns) MD simulations. The study showed that the residues in the core are less mobile than those at the edges, coincident in both simulations and NMR data. From the simulations, a highly dynamic salt bridge network was identified, and deemed responsible for the thermostability of the fibers. Regarding the hexapeptide VEALYL from insulin, Matthes et al. (2014) build different models of a fiber with nine protofibrils in a microcrystalline array, at pH 2.5, 4, and 7, obtaining different degrees of twisting. By comparing the predicted chemical shifts for the simulations with the experimental data, good agreement was observed, explaining how different conformations give rise to similar chemical shifts. Some residues have split values, completely in agreement with the ssNMR data.

Stopping amyloid fiber formation is an important goal in the clinic. An interesting case is that of alpha-synuclein (AS) and dopamine, an interaction that avoids amyloid fiber formation. AS is an IDP, so the use of unrestricted MD required clever choices of starting structures for the simulations. Dibenedetto et al. (2013) took six structures of AS, coming from an ensemble generated previously that was consistent with PRE measurements. These six structures cover ∼70% of the population. They docked to each of these six structures dopamine or one of five dopamine degradation products that could be physiologically relevant, yielding a total of 36 AS-dopamine adducts. Each free AS and adduct was simulated for 40 ns of standard MD, and for analysis they selected the frames with direct contacts of AS to dopamine or its derivatives. Comparison to SOFAST-HMQC NMR data (chemical shifts, RDCs) was good, and the preferred binding site was identified. All ligands cause an increase in secondary structure, helices and turns particularly, explaining the inhibitory effect on amyloid formation. Another interesting case is that of the formation of insulin fibers (Banerjee et al., 2013), which happen in patients with diabetes and in normal aging. Banerjee et al. (2013) describe a peptide called NK9 that delays the fibrillation process of insulin in a sub-stoichiometric ratio, affecting nucleation. They show with multiple techniques that NK9 binds an insulin trimer at low pH, and that the nucleus for fiber formation is an insulin monomer. With saturation transfer difference nuclear overhauser effect spectroscopy (NOESY) experiments they determine the binding site, and model it in a monomer, which was simulated for 100 ns to gain structural understanding of the interactions.

Enzymes with loops around the active site are common, and an important question is whether these loops help or hinder catalysis. Two studies of dimeric HIV protease show complementary approaches. Torbeev et al. (2011) carry out chemical synthesis of HIV to test D-amino acids and non-standard amino acids (such as Aib) in the flaps covering the active site. The conformational isomerization of two symmetry related flaps (residues 37–61) is correlated with the structural organization of the active site, and this step is rate limiting. The flaps sample conformations that depend on the backbone motion of G51, and exchange in the sub-ns and μs–ms timescales. S^2^ and car-purcell-meiboom-gill (CPMG) experiments show that the dynamics of the catalytic residues echo the dynamics of the flaps, and the coupling appears to be through water molecules. MD simulations of 300 ns of protonated or unprotonated D25 (the catalytic residue), and of L-alanine or D-alanine substitutions for G51 recover the interflap distances that were measured with electron paramagnetic resonance (EPR). Xia et al. (2013) build a MSM from multiple MD runs (20 ns × 20 ns) carried out in implicit solvent. The states are defined from the structural similarity of the flaps, resulting in four states. With the MSM they generate a stochastic trajectory of 10 μs, and this trajectory is the source of data for comparison with NMR. The MD runs are used to describe fast motions (100 ps), and the stochastic trajectory for slow motions (100 ns). In this work they propose new equations that describe the exchange between the four macrostates and the fast local motions in each. As in HIV protease, protonation of select residues is critical for activity in the mRNA decapping enzyme Dcp2 (Aglietti et al., 2013). The catalysis in this case is acid/base chemistry, mediated by Mg^2+^, which is bound to a loop that changes conformation during the catalytic cycle, so again a conformational change is important for activity. There are four nearby Es, one of which is the general base (E153), one is in the loop that changes conformation and binds Mg^2+^ (E198), and others bind Mg^2+^. Methyl-NMR and MD suggest that the loop with E198 is sensitive to the protonation state of E153, so they measured the pKa of E153 with NMR using ^13^C HSQC in a protein labeled in I, L, and V residues. They confirmed this residue as the general base, and showed that its protonation affects both the conformation and the dynamics of the loop, as I199 has linewidth changes, suggesting dynamics in the μs–ms timescale. They carried out MD simulations (100 ns) of the catalytic domain of Dcp2, with E153, protonated E153, and Q153. The observed structural and dynamic consequences of these modifications explain chemical shift displacements with pH, and the changes in dynamics.

The response of enzymes to their substrates or inhibitors is of great interest, as is the question of which of the motions of the free enzyme are relevant for catalysis. Calligari et al. (2012) explore the effect of 6-phosphogluconolactone on the enzyme 6-phosphogluconolactonase from *Trypanosoma brucei* (Tb6PGL), an interesting enzyme as it is a potential drug target. They measured the dynamics of both the apo and holo forms, analyzing relaxation data with the Model Free formalism of Lipari and Szabo (Lipari and Szabo, 1982). They found that residues far from the active site display differences in motion, but some close to the active site showed little or no difference. In order to understand these observations they carried out 77 ns MD simulations for both apo and holo forms. Those residues with converged order parameters showed good agreement between simulation and NMR data. The simulations showed that despite the lack of difference in motion in the backbone of the active site residues, side chains did show changes in motion without affecting the backbone. Changes in side chain rotamers are important for information transfer across proteins, and are the subject of two studies on the imidazole glycerol phosphate synthase (IGPS; Rivalta et al., 2012; Manley et al., 2013). This enzyme is large, therefore in order to study its dynamics, selective labeling of methyl groups in I, L, and V residues were done. The enzyme was studied in the apo form, in a binary complex with its substrate, and in a ternary complex with an allosteric effector too. Fast motions (ps–ns timescale) are similar for the three states, but μs–ms motions are different. In order to understand how these motions happen and change, four runs of 100 ns each, for the apo and the binary complex were run. A network analysis revealed the communities of residues that respond to the presence of the substrate, through changes in salt bridges and H-bonds, connecting the effector site to the active site. Another spectacular example is the study of allostery in the catalytic domain of protein kinase A (PKA; Masterson et al., 2012). NMR chemical shift titration experiments revealed local and long-range changes upon ligand binding, and the linear response to ligand concentration suggested an equilibrium between conformational states. Fast and medium timescale dynamics indicated an increase in motion upon nucleotide binding, and the synchronous motion of discrete regions leading to the opening and closing of the active site. The estimated rate constant between open and closed states coincides with the turnover constant, strongly suggesting that this motion is rate limiting and that nucleotide binding is the activator. Four MD simulations of 75 ns each were done for the apo PKA, PKA with nucleotide, and PKA with ATP and substrate or ATP and inhibitor. The simulations recover the trend in motion differences found experimentally. To understand better how this comes about, principal component analysis was done, with the first two modes accounting for more than half of the total motion of the protein. These modes correspond to opening and closing, and to a shearing motion of the domains. They propose that the nucleotide synchronizes motions in the enzyme, shifting the equilibrium to the closed state, in an allosteric process that changes both structure and dynamics.

Another scenario that is of increasing interest is the effect of crowding on both the native state and on unfolded states. Harada et al. (2013) explore the crowding of the villin headpiece, another iconic protein in simulations. They mix eight villin headpieces (36 residues) with four protein G (56 residues) as crowders, and modify the simulation cell size to change protein molar fractions from 12 to 43%, simulating them for 300 ns each. Experimentally they follow ^15^N labeled villin in the presence of unlabeled protein G, at a 17% molar fraction. In the simulations they find that the native state is prevalent in all conditions, but at higher crowding there is local unfolding. This unfolding is clearly different from that obtained at high temperatures or 8 M urea simulations, suggesting that the crowders modify the conformational landscape compared to dilute solution conditions. Protein G remains mostly unperturbed, and starts sampling near-native conformations only at the highest crowding condition. The changes in chemical shifts seen in the experiments are matched with the structural changes seen in the simulations, due to both inter and intramolecular events. At low concentration ion pairs are most important, but at higher concentrations both polar and hydrophobic interactions gain importance.

Sampling is always a concern, and an example of the types of care that must be taken is the analysis of internal dynamics in glutaredoxin by Allnér et al. (2015). Order parameters of amide and methyl groups obtained from NMR relaxation experiments are used to estimate the entropy of proteins, but these order parameters are estimated for each bond, lacking information on the correlation of motions of collections of these bonds (as in, for example, a whole alpha helix), resulting in an overestimated entropy. From the MD perspective, the convergence of the autocorrelation functions is essential to get reliable magnitudes for the order parameters, and this is a site-specific problem. Converged residues have good agreement with experimental values, regardless of the actual value of the order parameter, suggesting that the force fields do a good job. They find that sampling is better for many short runs (preferably started from different structures) than for a very long run, as even μs-long runs may not be converged.

A stringent test of both force fields and sampling is the simulation of unfolded proteins. Lindorff-Larsen et al. (2012) simulated acid-unfolded acyl-coenzyme a binding protein (ACBP) for 200 μs, starting from a fully protonated and high temperature unfolded state. The aim of this unrestrained simulation was to determine if CHARMM22* is capable of describing local residual structure and also the global structure of the unfolded ensemble. Qualitative agreement was obtained, but the simulated ensemble is more compact than what experiments suggest. NMR relaxation properties and PRE data can also be explained qualitatively through the presence of local structure, and residual alpha helical sections in the simulations correlate with those measured by NMR. Overall, the conclusion is that the simulation was too short to achieve quantitative agreement, but that the qualitative data are useful.

## Enhanced Sampling to Improve the Description of NMR Observables

Acknowledging that sampling is a pervasive problem, enhanced sampling protocols aim to solve this with different tricks. One of them consists on running multiple MD simulation replicas at different temperatures, and then exchanging replicas at specific time intervals according to a Metropolis criterion (REMD, see Figure 3). This allows the system to jump barriers connecting states easily, and therefore, to sample conformations efficiently. Grutsch et al. (2014) use this approach to study the native ensemble of a pollen allergen from birch (Bet v 1). These proteins store and transport small molecules, such as deoxycholate, in an internal cavity. How the ligand gets into the cavity is not known, but there are three possible entry sites that can be seen in crystal structures of members of this family. To make matters more interesting, Bet v 1 has many isoforms, these vary in immunogenicity despite having the same scaffold, and ligand binding also alters immunogenicity by stabilizing epitopes. In order to characterize how conformationally heterogeneous is apo Bet v 1, they ran REMD, with 32 replicas over 100 ns, keeping only the data of 300K for analysis; these runs show that the apo form samples conformers that resemble the holo protein, in agreement with the conformational selection model. They also ran 1 μs of standard MD for both apo and Bet v 1 with two deoxycholate molecules, starting from crystal structures. Experimentally they use pulsed-field-gradient methods to measure diffusion, as this is related to the diameter of the protein, and confirmed that ligand binding produces compaction of the protein. This coincides with the measures of the radius of gyration from the MD simulations with and without ligand. They also measured chemical shift displacements in the apo and holo protein with other ligands, finding very small changes; the largest changes are in the order parameters, which show how the protein becomes more rigid upon ligand binding. Medium timescale dynamics (μs–ms) is lost upon ligand binding. They argue that the freezing of these degrees of freedom could be important for the particular positioning of loops or epitopes involved in allergies.

The description of the ensembles for IDPs is, as noted above, particularly problematic. The presence of pre-structured motifs is important for specific binding, and their identification is a goal in bioinformatics. One approach is that of Szöllosi et al. (2014), who use discrete MD (DMD) and replica exchange in a collection of IDPs for which there is NMR data indicating the presence of alpha helices in particular regions of the proteins. DMD is a collision driven algorithm, with implicit solvent, and uses a force field based on chemistry at HARvard macromolecular mechanics (CHARMM). It is faster than standard MD, and it only calculates forces upon collisions, so the simulation time is measured differently. They analyzed secondary structure as a function of temperature, and as expected, it diminishes as temperature is increased. Overall, they can detect 45 out of the 65 pre-structured motifs in the set, indicating that the procedure is a good starting point to work with an uncharacterized protein. Designing inhibitors for IDPs is hard, because the binding sites can be moving targets. Jin et al. (2013) speak of ligand clouds around protein clouds, and use a fragment of oncogene cMyc(370–409) and a small inhibitor as a case example. Binding was measured with NMR, CD, and fluorescence. First they simulate the apo structure with REMD (30 replicas in implicit solvent) for 34.5 μs, and then with the ligand in explicit solvent for multiple 1 μs standard MD runs. They see that cMyc remains disordered, and the ligand binds at many locations; the ensembles were validated against proton and carbon chemical shifts. A non-ligand (another segment of cMyc) does not bind the ligand with the same efficiency, so this is not a case of blatant promiscuity.

Accelerated MD is another strategy to improve sampling. Here, the idea is to flatten out the free energy surface describing the conformational landscape of the protein, so barriers can be traversed easily. This is achieved by a boost term for the energy, and this boost is adjustable. Two examples of this approach (Salmon et al., 2012; Guerry et al., 2013) apply this to study the dynamics of an SH3 domain, compared to RDC. Salmon et al. (2012) accelerate their MD simulations to the point where the ensemble generated agrees best with the RDC data. Once this ensemble is defined, they calculate order parameters. Their data show that fast motions (ps–ns regime) are almost uniform in the SH3 domain, but those derived from RDCs show increased motions in the loops that interact with ubiquitin. They get the fast motions from standard MD runs seeded from the accelerated ensemble, a strategy reminiscent of the AS complexes with dopamine, described above (Dibenedetto et al., 2013). Guerry et al. (2013) take this approach further in a new method called SUPERNOVA, which is a multi-level AMD simulation. They oversample the SH3 domain to make sure they get all the sub-states; then they generate Boltzmann-weighted ensembles that agree with experimental data (RDCs obtained in various orienting media in this case). The chemical shifts calculated from these new ensembles agree much better with experimental data than the whole oversampled ensemble. The same idea, but using Monte Carlo simulations instead of AMD, was used before (Jensen et al., 2011) to study the tail domain of the measles virus nucleo-protein. With the flexible-meccano program, which uses volume exclusion and amino acid specific conformational preferences, they generated a huge set of conformers. They calculated chemical shifts and RDCs for the conformers, and selected the ensemble that described best the experimental data. They found that the domain adopts a dynamic equilibrium between unfolded conformations and helices of different lengths, some of which are retained upon capsid assembly.

Accelerated MD can also be used to break complexes and study their stability. A nice example of this strategy is that of Xing et al. (2014), applied to a phosphotransferase system, specifically the weak interaction between enzyme I and enzyme IIA-glucose (EI—EIIA). This interaction results in the phosphorylation of EIIA by EI. Chemical shift perturbation was used to map the interaction site and estimate the low Kd (>25 mM). Using these data and PRE data from specifically labeled samples, they built a model of the complex. This model was used with AMD, with the phosphate on EI or on EIIA, to test their stabilities, and also a mutant that increases the Kd was simulated. The mutant alters the electrostatics at the interphase, and results in a complex that does not dissociate.

## Restricted Simulations Using NMR Observables

This approach could be considered as the best way to complement the strengths of simulations and experiments. The drawback is that the restrictions are protein-specific, so what works for one need not work for others. Nevertheless, it is very powerful, as the following examples show. A natural problem for this approach is the description of IDPs or of the DSE of proteins. Esteban-Martin et al. (2013) used PRE to map tertiary interactions in AS, an IDP, using 11 PRE labels at different positions, and used these data to restrict a Monte Carlo simulation with a protocol called SCOOBE, where PRE are back-calculated for each Monte Carlo step, guiding the simulation. Once the sample is large enough, clustering is applied and representative structures are used to obtain detailed contact maps for the soluble protein. Curiously, some contacts are compatible with amyloid fibers but others are not, suggesting the need for a reorganization of the structure during the fibril formation process. Scaffolding proteins have disordered regions that sample compact states with residual secondary structure, and this is important for binding, as it reduces the entropy cost as discussed above. Krieger et al. (2014; Goto, 2014) use chemical shifts to characterize the unbound state of a peptide from scaffolding protein Gab2. The bound state is known from crystallography, and there the N-terminus is a polyproline sequence that binds in an extended structure to the Grb2 SH3 domain, while the C-terminus is a 3_10_ helix with positive residues. The free peptide shows beta regions and PP_II_ sites. They used MD simulations in explicit solvent with replica averaged backbone chemical shifts in CamShift as structural restraints, to recover the free energy landscape of Gab2. By mutating prolines to alanines, they can reproduce the ranking in binding affinity of the mutant peptides, just by looking at the amount of residual structure and the direct contacts. The conformational ensemble has two clear minima, one looking like the bound conformation and the other like a random coil, again as suggested by the conformation selection model. In stark contrast, simulations of calcium-loaded calmodulin (Ca-CAM), using carbon, nitrogen, and hydrogen chemical shifts as replica averaged constraints (Kukic et al., 2014), revealed that Ca-CAM in the absence of its ligands does not sample the bound conformation, at least down to 5–10% of the global population, arguing for induced-fit in this case. The ensemble generated agrees with data from RDC, PRE, SAXS, and FRET, only when all the chemical shift restraints are used. Furthermore, the behavior of the linker helix explains the behavior of the whole protein to a good approximation, and can also explain the effect of alanine mutations. In another exploration of the conformational landscape, Granata et al. (2013) applied metadynamics to fold the Ig-binding domain of protein G. Experimental chemical shifts were used as one of the seven collective variables that guided the sampling in explicit solvent. They ran 380 ns on seven parallel replicas, with replica exchange at room temperature. They found the native state as the lowest minimum on the landscape, and a compact intermediate state.

Regarding residual structure in denatured proteins Ozenne et al. (2014) characterize the acid unfolded state of ACBP, the same protein studied by Lindorff-Larsen et al. (2012). The idea is that the principle of minimal frustration implies that there is a bias in the DSE toward native-like structure, and this shows in secondary structure propensity and long-range contacts. There is evidence from chemical shifts, RDCs and PRE for denatured ACBP. In this paper they use PRE and mutants to obtain distance constraints for acid-unfolded ACBP and dissect whether contacts cause helices or the other way around. The mutants are at residues with high phi values, so they form part of the folding nucleus. They use flexible-meccano to generate a large pool of random coils, and then the ASTEROIDS program to select five representative ensembles that represent the experimental PRE data. The contact maps show both native and non-native contacts, and the hydrodynamic radius matches that expected from pulsed-field-gradient NMR data. It is more compact than a random coil, but not as compact as unrestricted MD simulations suggest. To establish cause and effect between contact formation and collapse, they perform MD folding simulations with a coarse-grained native-centric protein model, and identify one intermediate between the TSE and the native state. Notably, this intermediate was described previously by relaxation dispersion. Also, they find that early contacts bias the DSE, and the mutants allow for the folding pathway to be reconstructed. Long-range contacts make folding faster.

Abandoning disordered structures, the next ensemble of interest is that of folding or functional intermediates. Neudecker et al. (2012) studied an on-pathway intermediate for the folding of the Fyn SH3 domain, that participates in amyloid fiber formation. They used relaxation dispersion experiments to get structural information on this intermediate: chemical shift, RDCs, and chemical shift anisotropy. They then carried out REMD simulations restrained by these data for 4 ns, in order to construct a model of the intermediate, which was validated with H/D exchange NMR data. The intermediate is native-like, but it exposes a beta sheet with high propensity to form amyloid fibers, and this is suggested as the beginning of the aggregation pathway. This also hints that forming fibers requires just local unfolding to proceed. Following with more amyloidogenic precursors, Bemporad et al. (2012) track the precursor of fibers in acylphosphatase (AcP), by deleting the 11 amino acids at the N-terminus. They monitor the effect of the deletion with H/D exchange NMR experiments, and use these protection factor data to restrict MD simulations that annealed between 298 and 498 K to generate a large ensemble of structures in water and in Trifluoroethanol (TFE); the latter was used as a pro-fiber condition. The structure with TFE is native like, but it has larger root mean square distance or deviation (RMSD) fluctuations, an increased radius of gyration and less native contact, explaining its tendency to form fibers.

Our last two examples relate to functional dynamics. The first one (Nguyen et al., 2014) describes the motions of the acyl carrier protein (AcpP) in the context of the fatty acid synthase. They use chemical shift perturbation to understand how the protein binds its substrate and to a partner, and to validate the AcpP-FabA crystal structure obtained by cross-linking. In order to study the ms dynamics they combined AMD with experimental RDCs, tuning the boost energy until the best agreement with RDCs was obtained. Standard MD simulations were run to obtain order parameters in the ps–ns regime, starting from the accelerated ensemble. AcpP has similar fast dynamics when bound to its substrate (a fatty acid) or when bound to FabA. At the slower timescale the protein behaves differently with each ligand: AcpP exhibits a breathing motion, the interaction with FabA stabilizes an open conformation that allows the release of substrate and the capture by FabA. The last example (Kannan et al., 2014) describes the dynamics of the long loops of the HU protein and compares them to the conformation when bound to DNA. The bending of DNA is due to a clamping mechanism, using pre-sculpted hinge motions in free HU. They use methyl chemical shifts as replica-averaged restrains in MD simulations (CH3Shift), alone or in combination with backbone chemical shifts (CamShift) of the HU dimer and look at the range of motions. They also compare it to unrestrained MD simulations. Methyl restrains seem to be enough to guide the ensemble to a correct description of other NMR data not used as restrains, both structural and dynamic, but the best agreement is achieved by combining methyl and backbone chemical shifts. The conformational landscape of the “ears” of HU shows two minima, one with the “ears” pointing outward and another with collapsed “ears.” The DNA bound conformation lies between these two, in an intermediate. They propose that HU samples DNA segments in its extended state and then clamps on them, in transit to the collapsed state. Clamping controls the space available to DNA, while twisting wraps the “ears” along the minor groove. Curiously, the maximum observed twist matches the trajectory of the minor groove, so they propose that the propensity to bind DNA is programmed into the intrinsic motions of HU.

## Future Developments

From the cell surface and extracellular matrix all the way to the interaction of proteins with DNA, protein dynamics is central to function. Outstanding problems in MD simulations are inaccuracies in the force fields and insufficient sampling for unrestricted MD runs. We are optimistic in that both these problems will become smaller in the near future, as force field refinement continues and computers and algorithms for simulations are improved. This will also allow for the study of larger and more complex proteins. On the NMR side, a common complaint in the works we discussed is the risk of overfitting, and the complications arising from only looking at the backbone of the proteins. On the bright side, better instruments, novel labeling schemes and pulse sequences, the use of metals and other probes, and also the development of the equations to deal with multiple states simultaneously will continue to increase our capacity to study more complex motions in proteins. *In vivo* NMR holds great promise to study how proteins work in their natural milieu.

### Conflict of Interest Statement

The authors declare that the research was conducted in the absence of any commercial or financial relationships that could be construed as a potential conflict of interest.
